# Hepatitis C-related membranoproliferative glomerulonephritis in the era of direct antiviral agents

**DOI:** 10.1590/2175-8239-JBN-2020-0148

**Published:** 2021-02-12

**Authors:** Walid Ahmed Ragab Abdelhamid, Ali Shendi, Mahmoud Zahran, Eman Abd Elbary, Sawsan Fadda

**Affiliations:** 1 Zagazig University, Faculty of Medicine, Internal Medicine Department, Zagazig, Egypt.; 2 Zagazig University, Faculty of Medicine, Pathology Department, Zagazig, Egypt.; 3 Cairo University, Faculty of Medicine, Pathology Department, Cairo, Egypt.

**Keywords:** Virology, Immune System, Proteinuria, Sustained Virologic Response, Cryoglobulinemia, Mycophenolic Acid, Virologia, Sistema Imunitário, Proteinúria, Resposta Viral Sustentada, Crioglobulinemia, Ácido Micofenólico

## Abstract

Membranoproliferative glomerulonephritis (MPGN) is the most typical Hepatitis C virus (HCV)-associated glomerulopathy, and the available data about the utilization of direct-acting antivirals (DAA) in HCV-associated glomerulonephritis is inadequate. We evaluated the renal and viral response in two cases of HCV-related MPGN; the first caused by cryoglobulinemia while the second was cryoglobulin-negative. Both patients received immunosuppression besides DAA in different regimens. They achieved partial remission but remained immunosuppression-dependent for more than 6 months after DAA despite sustained virological response, which enabled safer but incomplete immunosuppression withdrawal. Both patients were tested for occult HCV in peripheral blood mononuclear cells and found to be negative. Hence, the treatment of HCV-related MPGN ought to be according to the clinical condition and the effects of drug therapy. It is important to consider that renal response can lag behind the virological response.

## Resumo

A glomerulonefrite membranoproliferativa (GNMP) é a glomerulopatia associada ao vírus mais típico da hepatite C (HCV), e os dados disponíveis sobre a utilização de antivirais de ação direta (AAD) na glomerulonefrite associada ao HCV são inadequados. Avaliamos a resposta renal e viral em dois casos de GNMP relacionados ao HCV; o primeiro causado por crioglobulinemia, enquanto o segundo era negativo para crioglobulina. Ambos os pacientes receberam imunossupressão além de AAD em diferentes esquemas terapêuticos. Eles alcançaram remissão parcial, mas permaneceram dependentes da imunossupressão por mais de 6 meses após os AAD, apesar da resposta virológica sustentada, que permitiu a retirada da imunossupressão mais segura, mas incompleta. Ambos os pacientes foram testados para HCV oculto em células mononucleares do sangue periférico e deram resultados negativos. Portanto, o tratamento do GNMP relacionado ao VHC deve ser de acordo com a condição clínica e os efeitos da terapia medicamentosa. É importante considerar que a resposta renal pode ficar aquém da resposta virológica.

## Introduction

Hepatitis C virus (HCV) can cause extra-hepatic manifestations including nephropathy and mixed cryoglobulinemia (MC). Glomerular diseases are the most common type of nephropathy related to HCV^1^. The foremost common variety of HCV associated glomerulopathy is immune-complex-mediated membranoproliferative glomerulonephritis (MPGN), which is linked to type II MC. It can also happen less commonly in the absence of cryoglobulinemia 2. Generally, patients with HCV infection have a greater chance of end-stage renal disease (ESRD) (4.3/1000 person-year), compared to patients without HCV infection (3.1/1000 person-year) 3.

Timely and effective management of HCV-related glomerular disease is paramount to improve the outcome. Various approaches have been recommended including immunosuppressive therapy (corticosteroids, cytotoxic agents, and monoclonal antibodies) and antiviral therapy. These regimens should be considered according to the level of proteinuria and kidney failure 4. In light of the Kidney Disease Improving Global Outcomes (KDIGO) suggestions, patients with minor or moderate kinds of HCV-associated glomerulonephritis with the steady renal condition and additional non-nephrotic proteinuria ought to be treated first with a direct-acting antiviral (DAA) regime. Patients with significant cryoglobulinemia or serious glomerular disease caused by HCV (i.e., rapidly developing renal dysfunction or nephrotic range proteinuria) should be considered for immunosuppressive therapy with or without plasmapheresis plus DAA therapies. Furthermore, patients, who do not improve with, or can't tolerate DAA have to be managed with immunosuppressive regimen 1.

Inadequate evidence exists on the usage of DAAs in addition to the safety and efficacy of using Mycophenolate Mofetyl (MMF) in patients with HCV-associated glomerular disease 5 ^,^ 6. Therefore, we intended to evaluate two cases of HCV-related MPGN who had acute worsening of renal function and were managed with immunosuppression and DAA treatment and to assess the effects of their management on renal function and HCV viral load.

## Case Studies

**Case 1:** A 42-year-old lady presented with a 1-month history of lethargy, low-grade fever, large joint arthritis, vasculitic lower limb skin rash, bilateral ankle edema, and facial puffiness.

Her creatinine increased from 1.8 mg/dL (eGFR = 34 mL/min/1.73 m^2^) to 2.3 mg/dL at presentation. She had proteinuria of 1275 mg/d and microscopic hematuria. Serology for HBV and HIV were negative with positive HCV antibodies and a viral load of 29,300 copies/mL. Cryoglobulins were positive with consumed C3 and C4 and positive rheumatoid factor. Kidney biopsy revealed MPGN with fibrocellular crescents.

She started steroid therapy (methylprednisolone 500 mg/d for 3 days, then prednisone 60 mg/day) and triple antiviral therapy (Sofosbuvir (400 mg od), Daclatasvir (60 mg od), and Ribavirin (RBV) (200 mg bid)). RBV was discontinued 3 weeks later due to declining hemoglobin and dual therapy continued for 12 weeks. Despite her creatinine decreased to 1.8 mg/dL (eGFR 34 mL/min/1.73 m^2^) during the 1st week, proteinuria increased to 7533 mg/d within 2 months. Monthly intravenous cyclophosphamide (800 mg) was prescribed to allow gradual steroid withdrawal.

**Figure 1:**
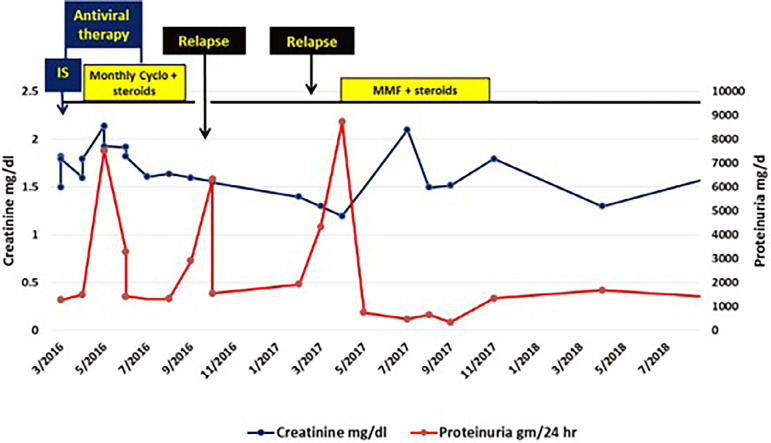
Graph showing creatinine and proteinuria change over follow-up period in case 1.

Creatinine and proteinuria then progressively declined to 1.6 mg/dL and 1.33 gm/d respectively and the patient achieved partial remission by the 6^th^ dose of cyclophosphamide (Figure 1). She could also achieve sustained virological response (SVR) with undetectable viral RNA 12 weeks after DAA therapy. However, when prednisone dose reduction was attempted to 10 mg/day, the patient had a relapse with a rise in proteinuria to 6.3 gm/day. This was 4 months after DAA therapy and 1 week after the last cyclophosphamide dose. Prednisone was then increased to 30 mg/day and MMF was instituted (1 gm bid). Proteinuria then declined to 1.56 gm/day and partial remission could be resumed. Five months later, she had another relapse also during steroid withdrawal. Occult HCV (HCV-RNA in peripheral blood mononuclear cells (PBMCs)) and cryoglobulins were undetectable when tested about 10 months after DAA therapy. After a follow-up period of 2 years and 6 months, the patient is on 5 mg/day prednisone and 1500 mg/day MMF.

**Case 2:** A 62-year-old lady presented with acute deterioration of kidney function while being evaluated for HCV treatment with DAA, associated with lethargy, pallor, extensive vasculitic eruption over her lower limbs, and marked splenomegaly. The patient was diagnosed with HCV in 2004 and had a skin biopsy for vasculitic rash revealing leukocytoclastic vasculitis. She was then treated with a combination of standard interferon and ribavirin, which failed to clear the virus. Her creatinine, which was 1.05 mg/dL, increased in 2013 to 1.7 mg/dL (eGFR= 33.4 mL/min/1.73m^2^), and continued to increase gradually thereafter.

In 2015, during the current presentation, creatinine increased from 2.05 mg/dL to 3.18 mg/dL over 5 months. She had proteinuria (1179 mg /day), microscopic hematuria, consumed C3, and C4 with undetectable cryoglobulins and rheumatoid factor. Serum HCV RNA was 4762872 IU/mL. Her kidney biopsy showed evidence of MPGN with thrombotic microangiopathy.

**Figure 2:**
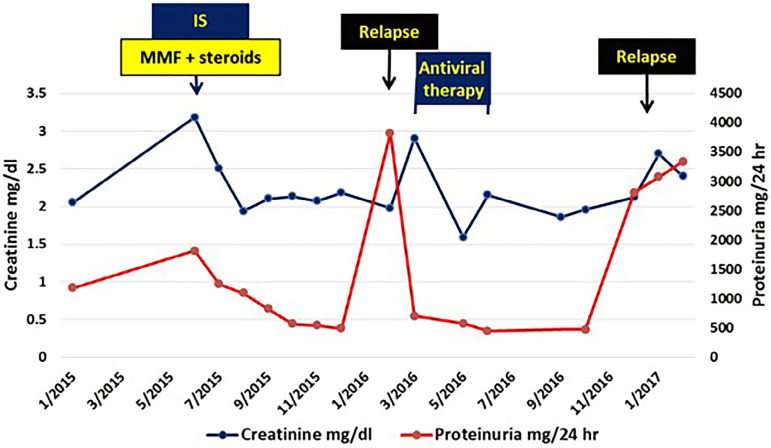
Graph showing creatinine and proteinuria change over follow-up period in case 2. IS: immunosuppression.

She started immunosuppression with tapering steroids (methylprednisolone 500 mg/d for 3 days then prednisone 60 mg/d) and MMF (1 gm bid) (Figure 2). Creatinine declined to 2.19 mg/dL (eGFR = 23.9 mL/) and proteinuria reached 1814 mg/day within the 1st month. Gradual steroid withdrawal was then implemented. MMF dose was reduced and then switched to mycophenolate sodium (720 mg bid) due to resistant diarrhea. Over the next 6 months, her creatinine remained stable with declining proteinuria to 488 mg/d. However, the patient had a relapse when steroid reduction was attempted to a daily dose of 15 mg prednisone. Her creatinine increased to 2.9 mg/dL and the proteinuria to 3823 mg/d, thus the previous daily dose of 20 mg was resumed and successfully kept her kidney function and proteinuria back to the previous values.

Nine months after starting immunosuppression, she started DAA (ombitasvir (12.5 mg), paritaprevir (75 mg), and ritonavir (50 mg) (2 tablets od) "Querivo^®^"). RBV (200 mg od) was added initially and discontinued after 2 months due to severe resistant anemia despite erythropoietin therapy, frequent blood transfusion, and RBV dose reduction to 200 mg EOD. The patient HCV RNA was undetectable by the end of DAA therapy and after 12 weeks.

After the antiviral therapy, the patient went in partial remission with stable kidney function (creatinine 1.96 mg/dL; eGFR 27.1 mL/min/1.73 m^2^), and proteinuria of 446 mg/day. She had a relapse when mycophenolate dose reduction was attempted 6 months after the antiviral therapy. However, careful steroid withdrawal was feasible until reaching alternating daily doses of 5 and 7.5 mg. Occult HCV was also undetectable when tested in PBMCs.

## Discussion

We report the cases of 2 patients with HCV-related MPGN; the first had MPGN caused by cryoglobulinemia while the second was cryoglobulin-negative. Both patients were indicated for treatment with immunosuppression besides the anti-viral therapy, and both could then achieve partial remission. However, the patients remained immunosuppression-dependent for more than 6 months after DAA despite achieving SVR which enabled safer but incomplete immunosuppression withdrawal.

The beneficial impact of viral clearance after antiviral therapy on proteinuria and kidney function in patients with HCV-related MPGN has been well established 7 ^,^ 8. However, clinical recovery with immunosuppression withdrawal is expected to be delayed after the viral response given that clearance of cryoglobulins can lag behind viral clearance by 4-6 weeks 9. Moreover, it is agreed among studies that the attainment of HCV RNA negativity does not always confer the resolution of HCV-related cryoglobulinemia. According to the case series reported by Sise et al. 8, cryoglobulin levels decrease reaching a nadir at a median of 4.6 months (range 22 days to13 months). Six of seven patients with HCV-related cryoglobulinemia complicated by MPGN achieved SVR and 3 of them had persistent cryoglobulin positivity. Patients with active GN experienced an improvement in eGFR and reduction in proteinuria after the successful treatment with DAA therapy. Six of the seven patients received immunosuppression before initiation of DAA therapy and only one received immunosuppression concurrent with antiviral therapy 8. Another case series reported five patients with HCV complicated by MC who had persistence of cryoglobulins with the resultant untoward clinical ramifications despite achieving SVR after having triple antiviral therapy. More interestingly, like in our first case, clearance of cryoglobulins did not necessarily ensure the resolution of clinical symptoms 10.

There is no clear mechanism to explain the lag in immunologic and/or clinical response behind the viral clearance. It can be argued that it takes some months for significant declines in cryoglobulin concentrations following successful antiviral therapy 11. This period may be longer in patients with more advanced chronic HCV who may have decreased ability to clear immune complexes 10. Another explanation is the presence of occult HCV infection despite negative serum viral load. A strong association was evident between occult HCV and immune-mediated GN when patients with negative serum HCV-RNA were tested for occult HCV-RNA in PBMNCs or in serum after ultracentrifugation 12. Similarly, the persistence of MC after HCV clearance is associated with the detection of occult HCV in PBMNCs 13. Interestingly, MPGN could even be induced by the persistence of HCV antigen in the kidney in patients with HCV-negative viral load and negative occult HCV in hematopoietic cells. In 3 patients with MPGN associated with MC and a history of HCV infection confirmed by the presence of serum anti-HCV antibodies with a negative viral load, no evidence of occult HCV infection was found in PBMCs and in the cryoprecipitate, but HCV-NS3 antigen was present in the kidney biopsy in one of them 14. Our patients were tested for occult HCV as a putative mechanism driving the ongoing immune activity and viral RNA was undetected in PBMNCs^15^.

We suggest that some forms of immune dysregulation can be responsible for the persistent immune activity after viral clearance. The presence of "point of no return" in the natural history of such lymphoproliferative disorder, with progressive independence from the etiologic agent cannot be excluded 13.

On the other hand, immunosuppression prescription for patients with severe forms of HCV-related glomerular disorders leads to improved outcome. In a prospective study, rituximab combined with Peg-IFN-a/ribavirin, in HCV-associated MC, had a better outcome than Peg-IFN-a/ribavirin alone 16. Patients with nephrotic-range proteinuria and/or rapidly progressive kidney failure or an acute flare of cryoglobulinemia should receive plasmapheresis, rituximab, or cyclophosphamide in conjunction with methylprednisolone and concomitant antiviral therapy 17. Choosing the appropriate immunosuppressive regimen should be individualized taking into account many factors: age, the severity of liver and renal involvement, extra-renal manifestations, any previous treatment, contraindications or adverse events, and the balance between immunosuppression and virus activity 6. Rituximab is recommended as the first line and cyclophosphamide as the alternative immunosuppressant 1 ^,^ 17, while the use of MMF was reported as an effective and safe alternative in few cases 5 ^,^ 6. In our first patient, we used cyclophosphamide as the initial immunosuppressive and then used MMF as the alternative when the patient had a late relapse. In the second, we used MMF as the initial immunosuppressive given her old age, prolonged history of chronic hepatitis C, long duration of kidney affection, and the presence of chronic anemia; all the clinical decision was made to avoid the high dose-cyclophosphamide regimen. In both patients, we experienced MMF as safe and effective with no major side effects except recurrent upper respiratory tract and urinary tract infections in the first patient, and diarrhea in the second, which settled by replacing MMF with mycophenolate sodium.

In conclusion, the management of HCV-related MPGN should be customized by the clinical seriousness and reaction to treatment. The renal reaction can delay after the virological reaction. Along these lines, it is critical to begin immunosuppression with or before the antiviral treatment in extreme cases and to maintain immunosuppression even after negative HCV viral load.
